# A developmental basis for stochasticity in floral organ numbers

**DOI:** 10.3389/fpls.2014.00545

**Published:** 2014-11-03

**Authors:** Miho S. Kitazawa, Koichi Fujimoto

**Affiliations:** ^1^Laboratory of Theoretical Biology, Department of Biological Sciences, Osaka UniversityToyonaka, Osaka, Japan; ^2^Research Fellow of the Japan Society for the Promotion of Science, Osaka UniversityToyonaka, Osaka, Japan

**Keywords:** floral development, organ number, ABC model, whorl, phyllotaxis, meristem, Statistics as Topic, Ranunculaceae

## Abstract

Stochasticity ubiquitously inevitably appears at all levels from molecular traits to multicellular, morphological traits. Intrinsic stochasticity in biochemical reactions underlies the typical intercellular distributions of chemical concentrations, e.g., morphogen gradients, which can give rise to stochastic morphogenesis. While the universal statistics and mechanisms underlying the stochasticity at the biochemical level have been widely analyzed, those at the morphological level have not. Such morphological stochasticity is found in foral organ numbers. Although the floral organ number is a hallmark of floral species, it can distribute stochastically even within an individual plant. The probability distribution of the floral organ number within a population is usually asymmetric, i.e., it is more likely to increase rather than decrease from the modal value, or vice versa. We combined field observations, statistical analysis, and mathematical modeling to study the developmental basis of the variation in floral organ numbers among 50 species mainly from Ranunculaceae and several other families from core eudicots. We compared six hypothetical mechanisms and found that a modified error function reproduced much of the asymmetric variation found in eudicot floral organ numbers. The error function is derived from mathematical modeling of floral organ positioning, and its parameters represent measurable distances in the floral bud morphologies. The model predicts two developmental sources of the organ-number distributions: stochastic shifts in the expression boundaries of homeotic genes and a semi-concentric (whorled-type) organ arrangement. Other models species- or organ-specifically reproduced different types of distributions that reflect different developmental processes. The organ-number variation could be an indicator of stochasticity in organ fate determination and organ positioning.

## 1. Introduction

Biological systems ubiquitously and inevitably exhibit stochasticity in traits from the molecular level to the multicellular and morphological level. The stochasticity in the numbers of protein molecules within single cells has been extensively analyzed in species ranging from bacteria to mammals (McAdams and Arkin, 1997; Elowitz et al., 2002; Paulsson, 2004; Sanchez and Golding, 2013) and follows a universal law: a gamma distribution whose parameters are given by the kinetic constants of transcription and translation (Cai et al., 2006; Pedraza and Paulsson, 2008; Taniguchi et al., 2010). Quantitative studies of stochasticity in molecule numbers have been applied to various functionalities of biological systems (Eldar and Elowitz, 2010; Balázsi et al., 2011), including drug resistance (Wakamoto et al., 2013), stress response (Locke et al., 2011), adaptation to fluctuating environments, signal amplification (Shibata and Fujimoto, 2005), experimental evolution (Sato et al., 2003; Ito et al., 2009), emergence of multicellular collective behaviors (Gregor et al., 2010), morphogen gradients (Houchmandzadeh et al., 2002; Bergmann et al., 2007; Gregor et al., 2007; Tkačik et al., 2008), pigmentation patterns (Nijhout, 2000), and others. Statistical noise at the molecular level can be transmitted to other levels of organization via biochemical reaction networks (Elowitz et al., 2002; Swain et al., 2002; Pedraza and van Oudenaarden, 2005; Shibata and Fujimoto, 2005). During multicellular development, the variation in molecular concentrations is either canalized (Waddington, 1942, 1959; Alvarez-Buylla et al., 2010), e.g., reduced (Manu et al., 2009), or amplified (Alim et al., 2012; Uyttewaal et al., 2012). Thus, molecular stochasticity is transmitted to macroscopic characteristics of organs and tissues, such as the domain size of gene expression (Manu et al., 2009) or the number of organs [e.g., body segments in vertebrates (Allen and MacDowell, 1940; Richardson et al., 1998) and Myriapoda (Kettle et al., 2003; Vedel et al., 2010), tentacles (Amui-Vedel et al., 2011), and floral organs in plants (Herrera, 2009)]. The developmental bases of stochasticity in the discrete traits, such as that in organ numbers, however, has been little examined in animals (Arthur and Farrow, 1999) and plants (Bachmann and Chambers, 1978). Do universal statistical laws govern the stochasticity appearing at the morphological level? If the answer is yes, then how do stochasticity at the molecular level and developmental properties regulate those laws?

**Figure 1:**
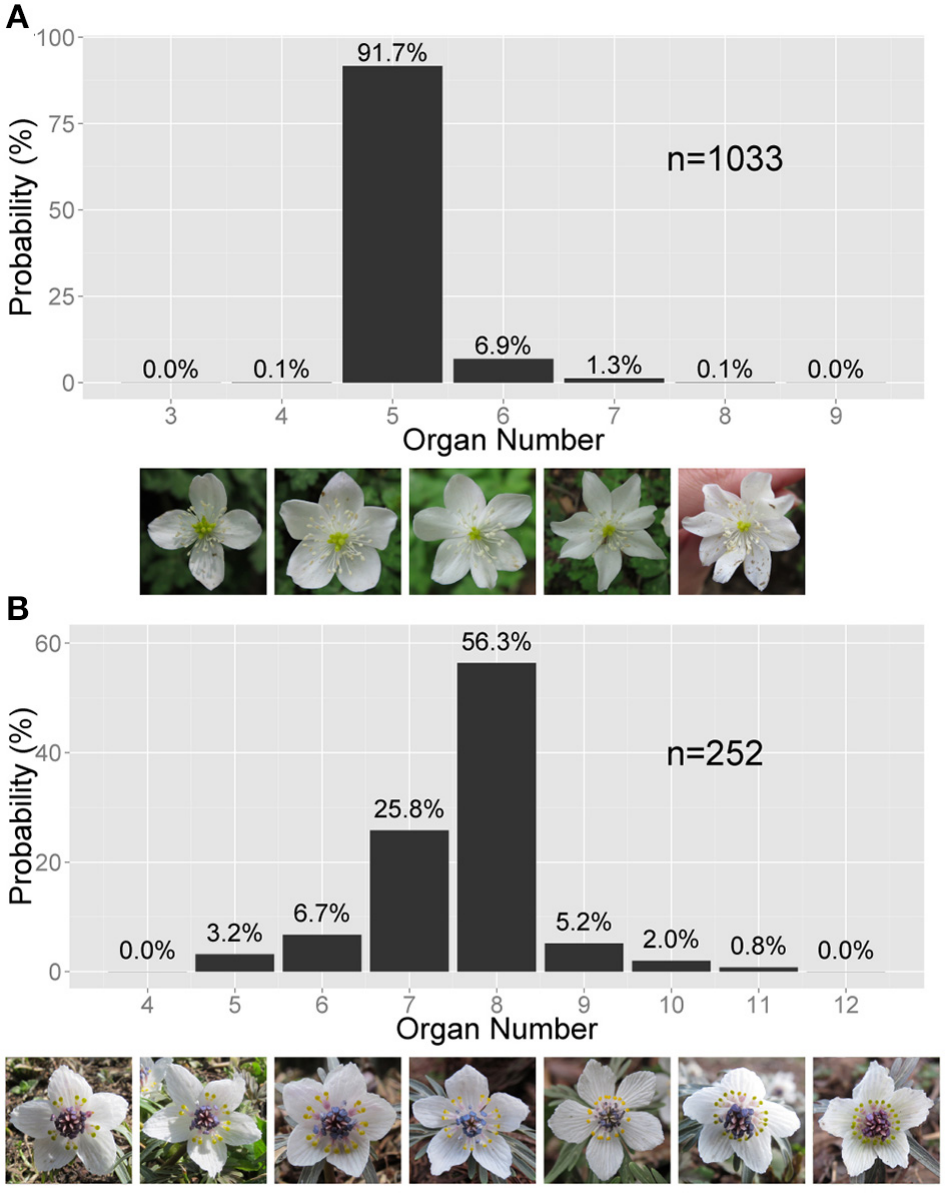
**Asymmetric variation in the Ranunculaceae floral organ number**. **(A)** Right-tailed variation in the tepal number in *Anemone flaccida*. The photographs show the flowers with four to eight tepals (the white, petal-like organs). **(B)** Left-tailed variation in the nectary number in *Eranthis pinnatifida*. The photographs show flowers with five to 12 nectraries (the yellow, forked organs). **(A,B)** The sample size *n* and the probability (%) are given on the bar chart.

Here, we focus on the discrete stochastic variation appearing in floral organ numbers. Although the floral organ number is a hallmark of eudicot species, it can distribute stochastically, even within an individual plant or a continuous population of a single species (Figure 1). The stochasticity has been quantified by the frequency distributions of floral organ numbers, including that of the floret numbers in the Asteraceae, in wild populations since the end of 19th century (de Vries, 1894; Ludwig, 1895). The most common distribution is positively skewed asymmetric distribution reflecting the fact that the organ number often increases from the mode but rarely decreases from the mode (Figure 1A; de Vries, 1894). Symmetric or negatively skewed asymmetric distributions (Figure 1B) were also observed (de Vries, 1894; Roy, 1963; Bachmann and Chambers, 1978). To account for the asymmetric distribution found in the Ranunculaceae, Pearson proposed the beta distribution (Pearson, 1895); however, there are three fundamental problems with that idea. First, the beta distribution requires continuous variables and is therefore not well suited to discrete organ numbers. Second, the beta distribution has not been examined in other species for over a century. Third, the beta distribution hardly gives a developmental underpinning. The floral organ numbers are determined during the initiation and fate determination of the floral organs. Scanning electron microscopic studies of the initiations of floral organs revealed that the sepal primordia initiate in sequential, helical order in the Ranunculaceae (Ren et al., 2010), the main target of the present paper, and in several other families such as the Caryophyllaceae (Lyndon, 1978) and the Oleaceae (Dadpour et al., 2011). These species exhibit considerable variation in floral organ numbers, as we will show. The identity of the organ primordia is determined after initiation by the so-called ABC genes (Coen and Meyerowitz, 1991). These two processes, the sequential initiation and subsequent determination of organ identity, are the candidate sources of the stochasticity in the floral organ numbers. The helical initiation order is similar to spiral phyllotaxis. Stochasticity in the angular and radial positioning of spiral phyllotaxis (Richards, 1951; Douady and Couder, 1996a) has been studied both experimentally (Peaucelle et al., 2007; Prasad et al., 2011; Besnard et al., 2013) and theoretically (Mirabet et al., 2012; Guédon et al., 2013). No model has been proposed, however, for the stochasticity in the organ positioning and the spatial expression pattern of fate determination genes during floral development.

We performed a review and statistical comparison of six hypothetical mechanisms for the stochastic determination of floral organ numbers in eudicots. We combined field observations, statistical analysis, and mathematical modeling to study the developmental basis of variation in floral organ numbers. The statistical selection of the best model to describe the observed variation in floral organ numbers clarified that a distribution based on a modified error function (the modified ERF) widely reproduced the asymmetric variation found in nature. The error function is derived from mathematical modeling of the floral organ positioning, and its parameters represent measurable distances on the floral bud morphologies. Moreover, the model predicts several mechanisms for the observed distributions (e.g., stochastic shifts in the expression boundaries of genes). The modified ERF model requires a semi-concentric organ arrangement (i.e., the whorled-type arrangement) to give an asymmetric distribution, whereas it does not require such an arrangement to give a symmetric distribution. Other models, i.e., the Gaussian, the Poisson, and the log-normal distributions, reproduced different types of variations species- or organ-specifically that reflect different developmental processes. The organ-number variation could be an indicator of stochasticity in organ fate determination and organ positioning during floral development.

## 2. Materials and methods

### 2.1. Plant samples

Populations of flowers were studied in natural and cultivated environments. The sampling of each floral population was limited both temporally (1–8 days) and spatially (diameter up to 100 m), because seasonal (Weldon, 1901) as well as geographical effects (Ludwig, 1901) on floral organ numbers can be significant. We also used published data sets. In total, we used 49 species mainly from basal eudicots (Ranunculaceae and Papaveraceae) and some from core eudicots (Asteraceae, Boraginaceae, Caryophyllaceae, Malvaceae, Oleaceae, Polemoniaceae, Primulaceae), which are listed with references in Table S1 of the Supplemental Data. The number of flowers/inflorescences *n* in each dataset is described at the top of each graph in Figures 1, **4** and in column *n* in Table S1. Detailed geographic and seasonal information is described in Table S1.

### 2.2. Statistical analyses

The fitting of the measured probability distribution to six statistics was determined using the non-linear least-square (NLS) method, where the probability of each organ number was a single data point. Because the organ number in each population does not distribute to a very large number of states (e.g., five states in Figure 1A), convergence is difficult to obtain using NLS. To improve the convergence, we adopted the Levenberg-Marquardt algorithm (Moré, 1978). For the Levenberg-Marquardt NLS fitting, we custom-designed a program using the R interface (http://www.r-project.org) with nlsLM function provided by the minpack.lm package (Elzhov et al., 2013) (Sample program available on request). The initial parameters were set arbitrarily to avoid parameter divergence during the NLS fitting.

One of the most popular and statistically rigorous criteria for selecting the best-fit model is the Akaike-Information Criterion (AIC), which is represented by the parameter number of the model *k* minus the natural logarithm of the maximum likelihood *L* (Equation 1; Akaike, 1974; Sakamoto et al., 1986; Burnham and Anderson, 2002).

(1) AIC=−2ln (L)+2k.

The AIC can be used to autonomously select the best-fit statistical distribution, which gives the minimum value of the AIC. When the number of states *M* denoting the number of the organ number with non-zero frequency (e.g., *M* = 5 in Figure 1A) is not very large compared with those of the parameters *k*, as in the present study, it is better to adopt the corrected AIC (AICc) given by

(2) $$\begin{array}{l} \text{AICc}=-2\ln\;(L)+\frac{2kM}{M-k-1}\\ \;=\text{AIC}+\frac{2k(k+1)}{M-k-1}, \end{array}$$

which must satisfy *M* > *k* + 1 and converges to the AIC at the upper limit of *M* (Sugiura, 1978). We computed the AICc for each combination of probability distribution and fitting function. Because the absolute value of the AICc does not have any meaning, we used ΔAICc, which is defined as the difference in AICc between a given model and the best model (Burnham et al., 2011), for ease of model comparison. Thus, the fitting function indicating ΔAICc = 0.0 is the best model, whereas models giving larger values are not as good. Generally, models with ΔAICc < 2.0 have the potential to be the best model, and those with ΔAICc < 7.0 cannot be easily rejected (Burnham et al., 2011).

### 2.3. Models of organ-number variation

Using the NLS algorithm, we fit the probability distribution of floral organ numbers to four continuous distributions (the standard Gaussian, the log-normal, the gamma, and the beta) and two discrete distributions (the Poisson and the modified ERF). We chose the four continuous models because the standard Gaussian distribution is the most basic distribution in statistics, the log-normal (Furusawa et al., 2005) and gamma (Taniguchi et al., 2010) distributions have been proposed as models for stochasticity in gene expression, and the beta distribution was previously suggested for organ-number variation (Pearson, 1895). Uncovering the developmental bases of the four continuous distributions of organ-number variation is a problem for future research, whereas the two discrete distributions are based on specific developmental processes.

#### 2.3.1. Continuous distributions

The probability density function of the standard Gaussian distribution is given by

(3) $$P_{sG}(X;\mu ,\sigma )=\frac{1}{\sqrt{2\pi }\sigma }\exp\left(-\frac{{\left(X-\mu \right)}^{2}}{2\sigma^{2}}\right).$$

**Figure 2:**
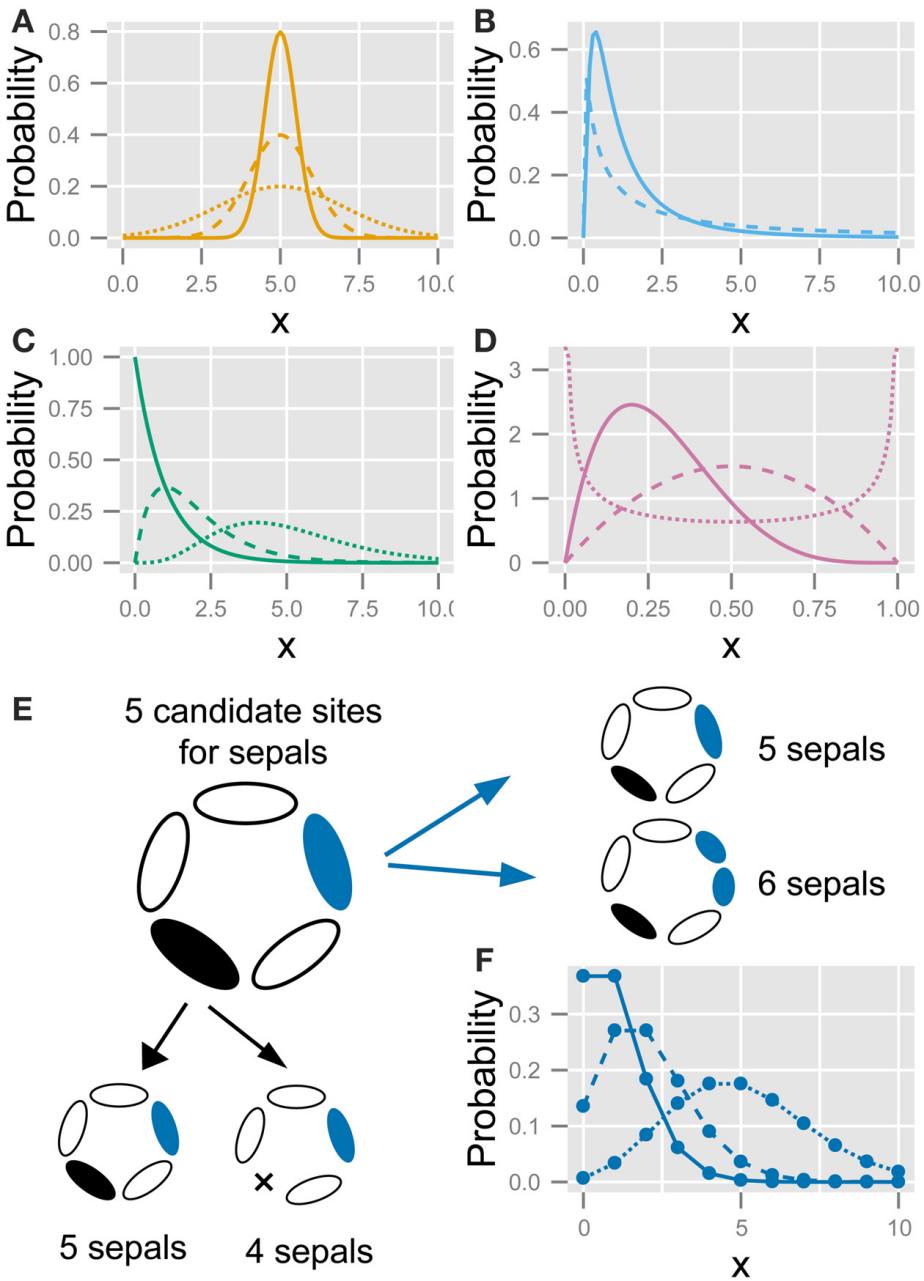
**The five statistics used to fit the variation in the floral organ number**. **(A)** Gaussian distribution (Equation 4). **(B)** Log-normal distribution (Equation 5). **(C)** Gamma distribution (Equation 8). **(D)** Beta distribution (Equation 8). **(E)** Schematic diagram of the developmental model for the Poisson distribution. There are five candidate sites for sepal development. Increasing variation: usually, all sites have one sepal, making the number of sepals five; some of the sites stochastically have two sepals (shown in blue), making the total number of sepals six; if two sites produce two sepals each, the total number of sepals is seven. Decreasing variation: some sites stochastically fail to develop a sepal (shown in black), causing the total number of sepals to decrease. **(F)** Poisson distribution (Equation 10). Different lines in **(A–D,F)** represent different parameter values.

This function exhibits a bell-shaped curve that is symmetric to the mean μ with standard deviation σ (Figure 2A). Although the values of the probability variable *X* are continuous, we assume that they represent the organ number.

The probability density function of the log-normal distribution is given by

(4) $$P_{ln}(X;\mu ,\sigma )=\frac{1}{\sqrt{2\pi }\sigma X}\exp\left(-\frac{{\left(\ln X-\mu \right)}^{2}}{2\sigma^{2}}\right).$$

This function represents a Gaussian distribution when *X* is on a logarithmic scale, but it is skewed to larger values of *X* on a linear scale (Figure 2B).

The probability density function of the gamma distribution, which is also skewed to larger values of *X*, is given by

(5) $$P_{\Gamma }(X;k,\theta )=\frac{X^{k-1}\exp\left(-X/\theta \right)}{\Gamma \left(k\right)\theta^{k}},$$

which has two parameters, the shape *k* (>0) and the scale θ (>0), and the gamma function Γ(*k*) (Figure 2C). The mean and standard deviation are *k*θ and $\sqrt{k}\theta$, respectively. The origin of the probability variable *X* = 0 in the log-normal and the gamma distributions can be shifted using another parameter *c*:

(6) $$P_{mln}\left(X;\mu ,\sigma ,c\right)=P_{ln}\left(X-c;\mu ,\sigma \right).$$

(7) $$P_{m\Gamma }\left(X;k,\theta ,c\right)=P_{\Gamma }\left(X-c;k,\theta \right).$$

The probability density function of the beta distribution is given by

(8) $$P_{\beta }(X;\alpha ,\beta )=\frac{X^{\alpha -1}{\left(1-X\right)}^{\beta -1}}{\text{B}\left(\alpha ,\beta \right)},$$

where B(α, β) is the beta function. The probability density function is not only skewed to either larger or smaller values of *X* but also bimodal, depending on the two shape parameters α and β (Figure 2D). Because the domain of the beta distribution is restricted to values between *X* = 0 and *X* = 1, in order to apply the beta distribution to floral organ numbers, the domain should be expanded between two real-number parameters *c_max_*, i.e., the maximum organ number, and the minimum organ number *c_min_* (*c_max_* > *c_min_*). By normalizing the factor *c_max_* − *c_min_*, the probability density function of the modified beta distribution can be represented by

(9) $$P_{m\beta }(X;\alpha ,\beta ,c_{max},c_{min})=\frac{P_{\beta }\left(\frac{X-c_{min}}{c_{max}-c_{min}};\alpha ,\beta \right)}{c_{max}-c_{min}},$$

where *X* denotes the organ number and $\frac{X-c_{min}}{c_{max}-c_{min}}$ is bounded by 0 and 1. We estimated this functional form of Equation 9 from Pearson's original paper (equation in the middle of p. 401 in Pearson, 1895).

#### 2.3.2. A developmental model of the Poisson distribution

Suppose that each flower has a special number *c* of candidate sites that usually grow one primordium but rarely have two primordia. When the probability of having two primordia is very low but not negligible (i.e., the average value of λ in Equation 10 below is on the order of unity or more and is given by *c* × *n* × *p*, where *p* is the probability that the rare event occurs and *n* is the number of counted flowers), the organ number satisfies the condition for the Poisson distribution. Bachmann and Chambers (1978) predicted this as the developmental source for the Poisson distribution of floral organ-number variation. If a candidate site can have one or two primordia, the distribution becomes right-tailed; whereas if a site can have one or no primordium, the distribution becomes left-tailed (Figure 2E). A stochastic increase in the number of primordia is reminiscent of reaction-diffusion-like patterning: a single concentration peak (e.g., a peak in phytohormone auxin concentration) preceding the emergence of a primordium sometimes splits into two primordia due to expansion of the space (Figure 2E, right panel). Such organ splitting was observed in *Abelia* leaves (Douady and Couder, 1996b) and tomato floral organs upon exposure to low temperature (Lozano et al., 1998). A stochastic decrease in the number of primordia can be induced by the fusion of two primordia that results no primordium in a candidate site (Figure 2E, bottom panel). The difference between the actual organ numbers and the mode *c* follows the Poisson distribution when the probability of a stochastic increase or decrease is very low but not negligible.

The probability of the Poisson distribution is given by

(10) $$P_{Po}(X;\lambda )=\frac{\lambda^{X}}{X!}\exp\;\left(-\lambda \right),$$

where the parameter λ corresponds to the mean (Figure 2F). By introducing the parameter representing the mode *c*, the equation is modified to

(11) $$P_{mPo}(X;\lambda ,c)=P_{Po}(X-c;\lambda ).$$

#### 2.3.3. A developmental model of the error function

**Figure 3:**
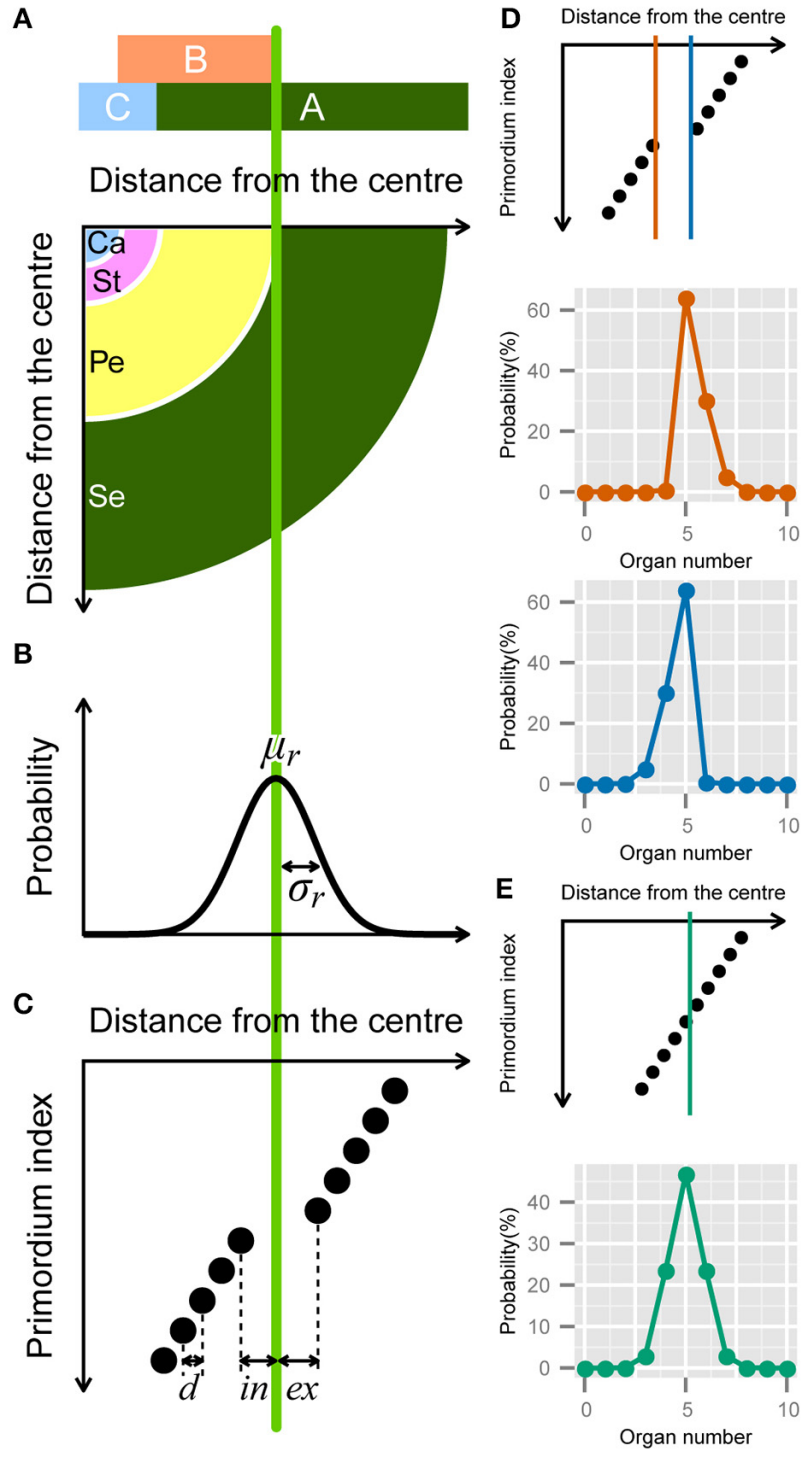
**Developmental model for the modified ERF distribution**. **(A)** Schematic illustration of the ABC model. A quarter of a flower separated into four regions (bottom panel) according to the expression of the ABC genes (upper panel). Se: sepals; Pe: petals; St: stamens; Ca: carpels. **(B)** The variation in the position of the expression boundary of the B gene follows a normal Gaussian distribution with average μ_*r*_ and standard deviation σ_*r*_ (Equation 12). **(C)** Schematic diagram of the modified ERF model. In a single whorl, all primordia have an identical radial distance *d* from the previous primordia. The distance gap between successive whorls is given by *ex* + *in*, where *ex* and *in* denote the distance from the average boundary μ_*r*_ to the last (innermost) primordium of the first whorl and the first (outermost) primordium of the second whorl, respectively, (Equation A2 in Supplementary Material). **(D)** *ex* > *in* causes a right-skewed distribution (orange), whereas *in* > *ex* causes a left-skewed distribution (blue). **(E)** If *ex* = *in* = *d*/2, the distribution of the organ numbers becomes symmetric.

Some of the stochasticity in floral organ numbers is induced by so-called homeotic transformations, i.e., the variations in the determination of floral organ identities (Goethe, 1790). For example, in a natural population of Ranunculaceae, the nectary-like or stamen-like narrow tepals sporadically appear (**Figure 5A**). Also, the increase in perianth organ number accompanied by disruption of the perianth/stamen boundary was observed experimentally by silencing the homeotic gene *APETALA3* paralog in *Nigella damascena*, Ranunculaceae (Gonçalves et al., 2013). Therefore, we constructed a model of homeotic transformations targeting the outer organs, such as the sepals, petals, and tepals, derived from the floral meristem. Floral organ identities are determined by homeotic genes, referred to as the ABC genes, which are expressed in a concentric manner (Coen and Meyerowitz, 1991; Figure 3A). For example, in the concentric region where only gene A is expressed (i.e., the region outside the expression boundary of gene B), the primordia differentiate into sepals. The homeotic transformations are observed even in an individual plant, indicating that it occurs non-genetically. The non-genetic homeotic transformations are explained by the variation of expression boundary of ABC genes, that is to say, when the expression boundary varies within a floral population, the number of sepals is variable. Similar variation in expression boundaries has been extensively studied in fruit fly *Drosophila* embryos. The boundary where the concentration of morphogens such as the Bicoid and Hunchback proteins exceeds a threshold value varies among individual embryos (Houchmandzadeh et al., 2002; Bergmann et al., 2007). In such cases, the threshold position can follow a Gaussian distribution if the concentrations of mRNA molecules, the concentration of morphogen degrading enzyme (which is usually proportional to enzyme degradation rate), and other molecular properties also follow a Gaussian distribution. Although quantitative studies of the ABC genes have not yet been reported, as an initial step, we assumed that the expression boundary positions of a gene determining floral identity follow a Gaussian distribution (e.g., B gene in Figures 3A,B). The probability density that the boundary is at radial position *r* is given by

(12) $$P_{gene}(r;\mu_{r},\sigma_{r})=\frac{1}{\sqrt{2\pi }\sigma_{r}}\exp\left(-\frac{{\left(r-\mu_{r}\right)}^{2}}{2\sigma_{r}^{2}}\right).$$

where *r*, μ_*r*_, and σ_*r*_ denote the radial distance from the meristem center and the average and standard deviation of the distance within the population, respectively (Figure 3B). The probability of having *X* organs is given by the integral of the probability that the boundary is located between the radial positions of *X*-th and *X* + 1-th primordia.

In addition to the boundary variation, we assume that the organs take on the semi-whorled arrangement that is widely observed in the Ranunculaceae (Ronse De Craene, 2010) and the Caryophyllaceae (Lyndon, 1978) (Figure 3C). The semi-whorl stands for the small variation among the radial positions within an apparent whorl. For simplicity, we assumed that the distances from the floral apex are regularly spaced within a whorl with an interval *d* (Figure 3C that represents a pentamerous whorl, i.e., the modal organ number *Mo* = 5, as an example). We defined the radial gap between two semi-whorls as *ex* + *in*, where the average position of expression boundary μ_*r*_ is located between the *Mo*-th and (*Mo* + 1)-th (e.g., the fifth and sixth when *Mo* = 5) primordia at distances *ex* and *in*, respectively. We used *d* as a scaling parameter (i.e., we set *ex_d_* = *ex*/*d*, *in_d_* = *in*/*d*, σ_*d*_ = σ/*d*). Thus, the probability of floral organ number *X* becomes (see the Supplementary Material for a detailed derivation)

(13) $$P_{er}\left(X;ex_{d},in_{d},\sigma_{d}\right)=\{\begin{array}{ll} \frac{1}{2}\text{erf}\left(\frac{ex_{d}+Mo-X}{\sqrt{2}\sigma_{d}}\right)-\frac{1}{2}\text{erf}\left(\frac{ex_{d}+Mo-X-1}{\sqrt{2}\sigma_{d}}\right) & \left(X<Mo\right)\\ \frac{1}{2}\text{erf}\left(\frac{in_{d}-(Mo-X)}{\sqrt{2}\sigma_{d}}\right)-\frac{1}{2}\text{erf}\left(\frac{in_{d}+(Mo-X+1)}{\sqrt{2}\sigma_{d}}\right) & \left(X>Mo\right)\\ \frac{1}{2}\text{erf}\left(\frac{ex_{d}}{\sqrt{2}\sigma_{d}}\right)-\frac{1}{2}\text{erf}\left(\frac{in_{d}}{\sqrt{2}\sigma_{d}}\right) & \left(X=Mo\right) \end{array},$$

where erf is the error function

(14) $$\text{erf}(z)=\frac{2}{\sqrt{\pi }}\int_{0}^{z}\exp\left(-z^{2}\right)dz.$$

The first line of Equation 13 (*X* < *Mo*) integrates the probability that the expression boundary is located within the exterior whorl, resulting in a decrease in the organ number. The second line (*X* > *Mo*) integrates the probability that the expression boundary is within the interior whorl. The third line (*X* = *Mo*) integrates the probability that the expression boundary is located within the gap between the two whorls. Because we assumed semi-concentric arrangement, there are several situations that the ERF model is unlikely to account for. Many of the Oleaceae species, except for *Jasminum* (Dadpour et al., 2011), show simultaneous initiation of all organs within a whorl, or even ring-like (early sympetalous) development of a whorl (Sehr and Weber, 2009). In such cases, the variation in the radial positions of the organs within a whorl should be negligibly small, differing from the assumption of the ERF model.

We analytically calculated the integrals using the NORM.DIST function in Microsoft Excel, which is the cumulative distribution function of the Gaussian distribution (Figures 3D,E). The three different forms of the function depending on the organ number (Equation 13) account for the two ubiquitous properties of floral organ-number variation (i.e., the asymmetry and the extraordinary mode probability). The skew to larger values of *X* becomes prominent as the difference *ex_d_* − *in_d_* increases (Figure 3D, orange line), whereas the skew to smaller values of *X* grows as *in_d_* − *ex_d_* increases (Figure 3D, light blue line). The modal probability becomes extraordinarily high as *in_d_* + *ex_d_* becomes larger than 1, indicating a semi-whorled arrangement. On the other hand, when *in_d_* + *ex_d_* is close to 1, indicating an equal radial distance between all successive primordia (*in_d_* + *ex_d_* = 1), as in spiral phyllotaxis (Höfmeister, 1868), the probability distribution becomes symmetric irrespective of *in_d_* and σ_*d*_ (Figure 3E). Thus, the modified ERF model predicts that asymmetric and symmetric organ-number variation indicate, respectively, the presence and absence of the pseudo-whorl.

## 3. Results

### 3.1. Variation of the floral organ number

We measured the frequencies of different floral organ numbers in populations of eudicots. By normalizing the frequency of each organ number by the population size (*n*), we reconstructed the probability distributions for 49 species, which were sampled by either ourselves or other authors (Table S1). As reported earlier (de Vries, 1894), the asymmetric distribution of floral organ numbers appears in many species (e.g., in the floral organs of the Ranunculaceae, Papaveraceae, and Caryophyllaceae and in the florets of the Asteraceae). Especially among the Ranunculaceae, most species exhibit asymmetric distributions.

There are two types of the asymmetric distribution: positively and negatively skewed distributions. In a positively skewed distribution, as in the tepals of *Anemone flaccida* (Figure 1A), the organ number often increases from the mode *Mo* but rarely decreases. In a negatively skewed distribution, as in the nectaries (located in the second whorl; petal-derived) of *Eranthis pinnatifida* (Figure 1B), the organ number often decreases from the mode *Mo* but rarely increases. In most cases, the asymmetric distribution is species-specific and organ-specific, and follows either of the two types. For example, *A*. *flaccida* tepals have a right-tailed distribution, not only in our observations made at different locations but also in previously published data (Ohno, 1991), whereas *E*. *pinnatifida* nectaries sampled in various locations have a left-tailed distribution (Table S1). In addition, the probability of the modal organ number is extraordinarily high in the floral populations compared with that in the Gaussian distribution, indicating the robustness of particular organ numbers. The extraordinary probability of the modal number is statistically represented by the high positive value of kurtosis, which the standard Gaussian distribution cannot account for (Sokal and Rohlf, 2012). Hence, another statistical law is necessary for both the asymmetry and the extraordinary mode manifested in the floral organ-number variation.

### 3.2. Selection of the best statistical model of the floral organ-number variation in Ranunculaceae

**Figure 4:**
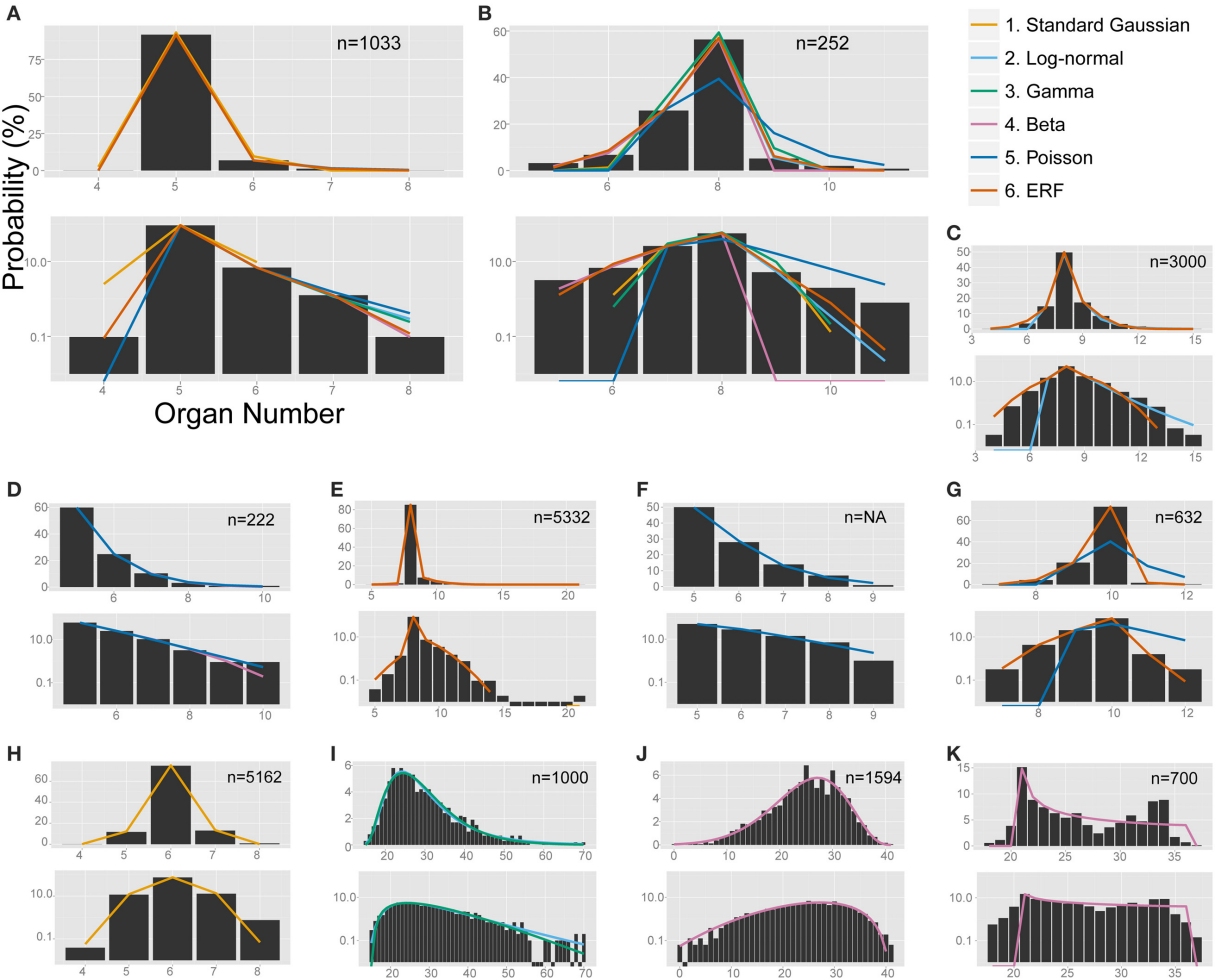
**The non-linear least-square fitting of the distribution of eudicot floral organ numbers**. Each of the six models (see the legend for color definition) is plotted in **(A,B)** whereas only the specified distributions are plotted in the other panels. In each panel, upper and bottom graph are linear and semi-log plot, respectively. ΔAICc values are shown in Table S4. **(A)** *Anemone flaccida* tepals. **(B)** *Eranthis pinnatifida* nectaries. **(C)** *Ranunculus ficaria* petals (Ludwig, 1901), fit best by the modified ERF distribution based on the ΔAICc, although the right tail follows the log-normal distribution. **(D)** *Ranunculus bulbosus* petals (de Vries, 1894), originally fit by the modified beta distribution by Pearson (1895), but better fit by the Poisson distribution. **(E)** *Sanguinaria canadensis* petals (Spencer, 1944), fit best by the modified ERF distribution. **(F)** *Microseris pygmaea* × *Microseris bigelovii* pappus parts (Bachmann and Chambers, 1978), best fit by the Poisson distribution. **(G)** *Microseris pygmaea* pappus parts (Bachmann and Chambers, 1978), best fit by the modified ERF and fit not as well by the Poisson distribution. **(H)** *Nyctanthes arbor-tristis* petals (Roy, 1963), fit best by the standard Gaussian distribution. **(I)** *Sanguinaria canadensis* ovules (Harris, 1910), fit well by the log-normal and gamma distributions. **(J)** *Hibiscus syricacus* seeds (Harris, 1911), best-fit by the modified beta distribution. **(K)** *Leucanthemum vulgare* ray florets (Ludwig, 1895), representing an example of a bimodal distribution fit by the beta distribution.

To find the best model for each pattern of floral organ-number variation and to elucidate whether there is any common law that unifies the patterns, we performed non-linear least-square fitting of each data set containing more than five states (histogram in Figure 4) to six theoretical probability distributions [i.e., the standard Gaussian (Equation 3), modified log-normal (Equation 6), modified gamma (Equation 7), modified beta (Equation 9), modified Poisson (Equation 11), and the modified ERF (Equation 13)]. For each data set, the best-fit distribution was selected by the AICc (Equation 2), which determines the best-fit distribution even when the number of parameters differs among the fitting functions (Akaike, 1974; Sugiura, 1978). In many cases, the ranking of the models based on the ΔAICc values were reproducible among different data sets representing the same organ in a given species (Table 1; see the Materials and Methods section for definitions of AICc and ΔAICc).

**Table 1:** **The reproducibility of ΔAIC among populations of two Ranunculaceae species: the tepals of *Anemone flaccida* (An.fl) and the nectaries of *Eranthis pinnatifida* (Er.pi)**.

**Species**	**Place/ref**	***n***	**Gauss**	**LogNormal**	**Gamma**	**Beta**	**Poisson**	**ERF**
An.fl	Mt. Ibuki	2519	^***^ 24.98	^*^ 1.85	^**^ 2.00	32.06	^***^ 8.35	^***^ 0.00
An.fl	Mt. Ibuki	2445	^***^ 15.03	^***^ 8.21	18.94	NA	^***^ 0.00	^**^ 2.02
An.fl	Mt. Ibuki	1033	^***^ 29.72	^**^ 21.32	^**^ 18.78	NA	^***^ 7.30	^***^0.00
An.fl	Mt. Kongo	1717	^***^ 15.63	31.51	^**^ 18.95	NA	^***^ 22.82	^***^ 0.00
An.fl	Mt. Kongo	671	^***^ 19.44	^***^ 9.84	^***^ 0.32	23.04	^***^ 29.16	^***^ 0.00
An.fl	Mt. Kongo	1528	^***^ 35.79	42.60	^*^ 42.19	NA	^***^ 36.45	^***^ 0.00
An.fl	Mt. Kongo	1384	^***^ 21.84	12.12	^***^ 11.67	41.55	^***^ 22.24	^***^ 0.00
An.fl	Mt. Kongo (sum)	5702	^***^ 23.56	17.70	^**^ 17.58	47.57	^***^ 27.75	^***^ 0.00
An.fl	Sasayama City	355	^***^ 9.14	15.13	13.35	NA	^***^ 0.00	^**^ 10.97
An.fl	Hanno City Ohno, 1991	1624	^***^ 20.76	^***^ 1.53	^***^ 3.60	17.70	^***^ 20.63	^***^ 0.00
Er.pi	Maibara City	47	^***^ 2.68	20.38	20.38	NA	12.67	^**^ 0.00
Er.pi	Maibara City	165	^***^ 0.00	10.29	10.34	17.58	6.74	9.67
Er.pi	Maibara City	153	^***^ 16.15	26.73	26.73	48.16	23.58	^**^ 0.00
Er.pi	Maibara City	252	^***^ 7.86	11.99	15.59	22.05	^**^ 19.71	^***^ 0.00
Er.pi	Maibara City	98	^***^ 1.18	6.85	7.12	^***^ 5.32	^***^ 9.10	^**^ 0.00
Er.pi	Sasayama City	184	^***^ 0.02	3.08	2.58	0.00	7.02	2.62
Er.pi	Sasayama City	127	^***^ 0.00	10.36	10.38	39.37	^***^ 5.50	9.63
Er.pi	Sasayama City	239	^***^ 11.01	18.54	18.84	20.58	20.74	^***^ 0.00

Some of the AICcs for the beta distribution were not available (NA) due to a smaller number of possible states (Equation 2). The p-values of the parameters are:

*** *<0.01*,

** *<0.05*,

* *<0.1*.

#### 3.2.1. The modified ERF model could account for an extraordinarily high mode and asymmetric tails on both sides of the distribution

The modified ERF model was the best fit for the outer floral organs in half (50/99) of the Ranunculaceae data sets [*Anemone flaccida* tepals; *A*. *hupehensis* var. *japonica* tepals; *A*. *narcissiflora* ssp. *nipponica* tepals; *A*. *nemorosa* tepals (Yule, 1902); *A*. *nikoensis* tepals; *A*. *raddeana* tepals; *Eranthis hyemalis* tepals and nectaries (Salisbury, 1919); *E*. *pinnatifida* tepals and nectaries; *Hepatica nobilis* var. *japonica* tepals; *Ranunculus arvensis* sepals and petals (Burkill, 1902); *R*. *bulbosus* petals (de Vries, 1894); *R*. *cantoniensis* petals; *R*. *ficaria* (syn. *Ficaria verna*) sepals and petals (Babington, 1834; Ludwig, 1901; Macdonell, 1903; Salisbury, 1919); *R*. *japonicus* petals; *R*. *parviflorus* petals (Salisbury, 1931); *R*. *repens* sepals and petals (Pledge, 1898; Salisbury, 1973); *R*. *silerifolius* petals; *Trollius europaeus* tepals and nectaries (Schöffel, 1932); the results are shown in Table 2 if there are more than four data sets for the species], especially in the *A*. *flaccida* tepals (8/10 data sets; Figure 4A), *E*. *pinnatifida* nectaries (5/8 data sets; Figure 4B), and *R*. *ficaria* petals (11/18 data sets; Figure 4C). Other models were selected less frequently: Poisson (24/99 data sets), standard Gaussian (10/99 data sets), log-normal (6/99 data sets), beta (5/99 data sets), and gamma (4/99 data sets). The features of the variation that were best fit by the ERF were the high modal probabilities and the two-sided asymmetric tails, which the other five models could not simultaneously account for. We have not yet concluded, however, whether the tails of all the data sets follow the modified ERF distribution, because some of the data sets have too few organ-number states *M* (e.g., *M* = 5 in *A*. *flaccida* tepals; Figure 4A), and others exhibit a linear right-side tail on the semi-log plot, which seems to fit a log-normal distribution more closely (e.g., *R*. *ficaria* petals; Figure 4C).

**Table 2:**
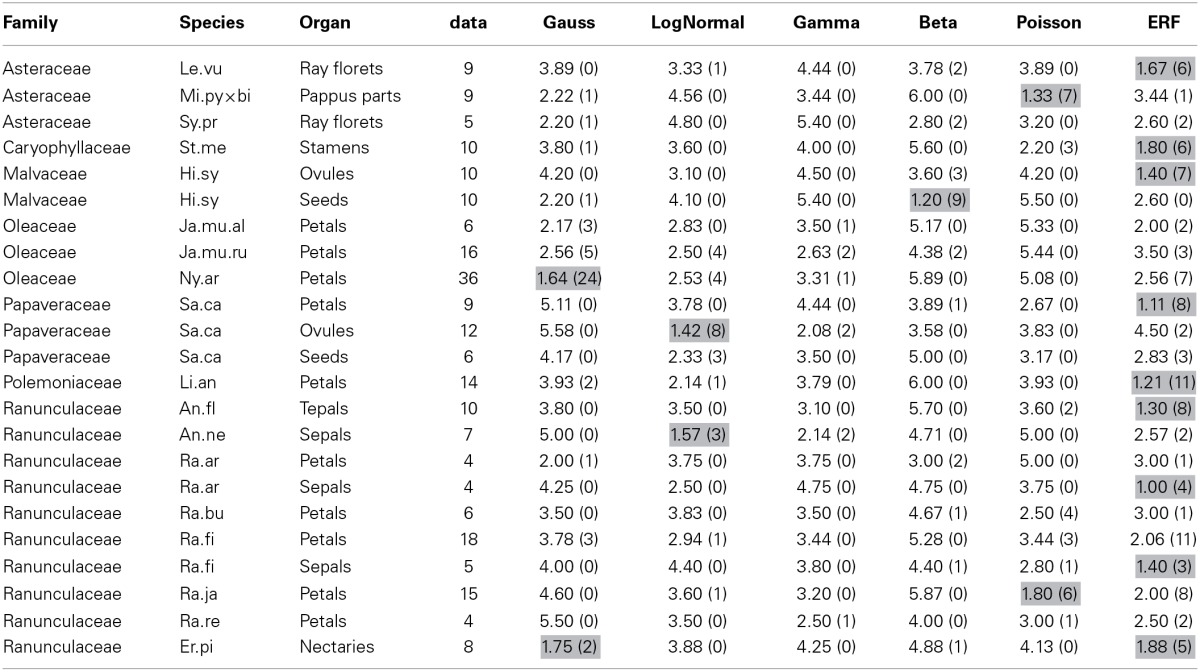
**The average rank score for each of the six models based on the ΔAICcs among the data sets for each combination of species and organ-type**.

For each data set, the best model was ranked 1, whereas the worst model and models that could not be scored were ranked 6. The number in ( ) denotes the number of data sets for which the given model was ranked 1. Le.vu: Leucanthemum vulgare from Ludwig (1895), Pearson and Yule (1902), and Tower (1902); Mi.py×pi: Microseris pygmaea×Microseris bigelovii from Bachmann and Chambers (1978); Sy.pr: Symphyotrichum prenanthoides from Shull (1902); St.me: Stellaria media from Burkill (1895); Hi.sy: Hibiscus syricacus from Harris (1911); Ja.mu.al: Jasminum multiflorum var. alba from Roy (1963); Ja.mu.ru: Jasminum multiflorum var. rubscens from Roy (1963); Ny.ar: Nyctanthes arbor-tristis from Roy (1963); Sa.ca: Sanguinaria canadensis from Spencer (1944) and Harris (1910); Li.an: Linanthus androsaceus from Huether (1969); An.fl: Anemone flaccida; An.ne: Anemone nemorosa from Yule (1902); Er.pi: Eranthis pinnatifida; Ra.ar: Ranunculus arvensis from Burkill (1902); Ra.bu: Ranunculus bulbosus from de Vries (1894); Ra.fi: Ranunculus ficaria from Babington (1834), Ludwig (1901), Weldon (1901), Macdonell (1903), and Salisbury (1919); Ra.ja: Ranunculus japonicus; Ra.re: Ranunculus repens from Salisbury (1973) and Pledge (1898). The gray cells indicate the averaged rank is less than two.

#### 3.2.2. Poisson distribution accounted for the one-sided distribution of petal numbers in Ranunculus better than the beta distribution

The Poisson distribution was the second dominant model (best fit for 24/99 data sets) for the Ranunculaceae perianth organs. Especially for the petal numbers in genus *Ranunculus* (excluding *R*. *ficaria*), the Poisson distribution was selected as often (12/32 data sets) as the ERF model (12/32 data sets). Pearson (1895) applied the beta distribution to petal-number variation in *R*. *bulbosus*. We statistically revisited the six original data sets of *R*. *bulbosus* measured by de Vries (1894), which include two natural populations, one cultivated population, and three populations selected over several generations for greater petal number by the author. Contrary to previous results (Pearson, 1895), four data sets [i.e., the two natural populations (including the data Pearson, 1895 used), the cultivated population, and one population selected for a petal number greater than five] had a mode of five and a tail on only the right side, which was best fit by the Poisson distribution (Figure 4D). The other two populations, which had a mode between eight and ten and tails on both sides of the distribution due to severe selection for more than nine petals, were best fit by the ERF and beta distributions, respectively. In addition, the Poisson distribution was often the best fit for variation in *R*. *japonicus* petals, which have five as the mode; the Poisson was the best fit for 6/15 data sets, and the modified ERF was the best fit for 8/15 data sets. In contrast, variation in *R*. *ficaria* petals, which have eight as the mode, was more often best fit by the modified ERF dominance model (11/18 data sets) compared with the Poisson infrequency distribution (3/18 data sets), as described above (Figure 4C). Transitions between the Poisson distribution that represents duplication of some primordia and the ERF distribution that represents homeotic variation may occur during evolutionary changes of the modal petal numbers in genus *Ranunculus*.

### 3.3. Some notes on other clades

#### 3.3.1. The ERF model in other clades

The ERF model was the best model for many other data sets in which the modal number has an extraordinarily high probability (i.e., the modal number is very stable), and the organ number varies on both sides of the mode. On the semi-log plot of *Sanguinaria canadensis* (Papaveraceae) petals, as the organ number moves away from the mode, the probability gradually decreases in a manner similar to the parabolic function that is consistent with the Gaussian distribution (Figure 4E). The extraordinarily high modal probability is inconsistent with the Gaussian distribution, however, and was best fit by the modified ERF distribution both in appearance (Figure 4E) and in terms of ΔAICc (Table 2); the upwardly convex tail of both the smaller and larger organ numbers in the semi-log plot could not be fitted by the other four models. We also found that the modified ERF model best fit the organ numbers in core eudicots, for example in *Linanthus androsaceus* (Polemoniaceae) petals (Huether, 1969; 11/14 data sets) and in *Stellaria media* (Caryophyllaceae) stamens (Burkill, 1895; 6/10 data sets). These results indicate that the ERF model widely accounts for perianth organs, and even for stamens, in core eudicots.

#### 3.3.2. The Poisson distribution fits the distributions of pappus part numbers of hybrid of *Microseris*, Asteraceae

The Poisson distribution of floral organ numbers was originally proposed for interspecific hybrids of *Microseris* (Bachmann and Chambers, 1978). Because the modal organ (pappus parts) numbers of the two parental species are different, the organ number of the hybrids varies between the two parental modes (Bachmann and Chambers, 1978). We statistically tested the distributions of pappus part numbers in nine of the interspecific hybrid populations of the genus *Microseris* published by Bachmann and Chambers (1978). Consistent with the earlier studies, seven of nine data sets for the hybrids generated by *M*. *pygmaea* × *M*. *bigelovii* crosses were best fit by the Poisson distribution (Figure 4F). On the other hand, all of the pappus-number distributions in the three genuine species *M*. *douglasii, M*. *lindleyi*, and *M*. *pygmaea* best fit the modified ERF distribution rather than the Poisson distribution (the ΔAICc for the Poisson were 23.53, 31.70, and 50.01, respectively; Figure 4G), suggesting that the Poisson distribution is the best model only for hybrid populations whose parental species have different modal organ numbers in Asteraceae pappus parts.

#### 3.3.3. Different developmental processes are reflected in the variation of Oleaceae petal numbers

In Oleaceae flowers measured by Roy (1963), more than half of the data sets best fit the standard Gaussian distribution (Table 2; Figure 4H). Especially in *Nyctanthes arbor-tristis*, the standard Gaussian distribution gave the lowest ΔAICc value for two thirds (24/36) of the data sets. In *Jasminum multiflorum*, the Gaussian distribution was the best fit for less than half of the data sets (3/6 in var. *alba* and 5/16 in var. *rubscens*), but the standard Gaussian was still more often the best fit than any of the other models (one gamma and two ERF in var. *alba*; four log-normal, two gamma, two beta, and three ERF in var. *rubscens*). The dissociation from the standard distribution correlates with the modal number, which is 6 in *N*. *arbor-tristis*, 8 in *J*. *multiflorum* var. *alba*, and 10 in *J*. *multiflorum* var. *rubscens*. The dissociation is partially due to the right tail of the distribution being longer than the left tail in some of the *J*. *multiflorum* var. *rubscens* data sets, in contrast to the symmetric variation in *N*. *arbor-tristis* and *J*. *multiflorum* var. *alba*, implying two mechanisms, symmetric variation with a smaller mode and asymmetric variation with a larger mode, respectively. The two mechanisms can be derived from differences in the developmental process between the two genera. The major differences are the number and the initiation order of the sepals, which can affect the petal development, because the petals develop adjacent and subsequent to the sepals. In addition to the four coincident sepals common in Oleaceae flowers, the *N*. *arbor-tristis* flower develops two extra sepals (Sehr and Weber, 2009). On the other hand, the sepal initiation in genus *Jasminum* is much more complicated. *J*. *nudiflorum* develops six sepal primordia in the same order as *N*. *arbor-tristis*, whereas *J*. *fruticans* shows pentamerous helical initiation but stochastically develops four or six sepals (Sehr and Weber, 2009; Dadpour et al., 2011). Thus, there are three patterns of Oleaceae sepal initiation: the simultaneous initiation of four sepals, the extra two sepals (4 + 2) found in *N*. *arbor-tristis* and some *Jasminum*, and the sequential helical initiation of five sepals similar to that in the basal eudicots (Ren et al., 2010). The inferiority of the ERF model is consistent with our assumption, because the first two patterns (i.e., the simultaneous and 4 + 2 initiation patterns) are unlikely to show a semi-concentric arrangement. The standard-Gaussian-like variation can be a characteristic of the 4 + 2 initiation pattern, whereas the dissociation from the standard Gaussian distribution in genus *Jasminum* can indicate the other patterns.

#### 3.3.4. Different organs followed different statistical laws in the same species

The best-fit models differed not only among the species, genera, and families described above but also among the organ types. In order to demonstrate that, we performed ΔAICc-based model selection for the distributions of three organ types in the same species (i.e., the petals, ovules, and seeds of *Sanguinaria canadensis*; Harris, 1910; Spencer, 1944; Table S2; Figures 4E,I). As the average ranks of each model for each organ show (Table 2), different models were selected for the petals and the ovules. For the petal whorls, seven out of eight data sets had the lowest ΔAICc with the modified ERF distribution; whereas for the ovules, eight out of 12 data sets were best fit by the log-normal distribution. For the seeds, three data sets were best fit by the modified ERF distribution, and the other three data sets were best fit by the log-normal distribution, although the parameters were not strongly supported (see the asterisks in Table S2). Those results support our ERF model for the perianth organs and suggest that we can distinguish different developmental processes in *S*. *canadensis* by organ-number variation. Petals form on dome-shaped floral buds in centripetal order: in typical *S*. *canadensis* flowers with eight petals, two sepal primordia first arise simultaneously, and two outer-petal primordia and two inner-petal primordia follow in a decussate manner, followed by four additional petals initiating at alternate positions relative to the four precedent petals (Lehmann and Sattler, 1993). On the other hand, the ovules form on the insides of cylindrical gynoecial primordia, although the process has not been studied in detail (Lehmann and Sattler, 1993). The number distributions of the ovules do not have extraordinarily high modal probabilities, contrary to those of the petals. For such distributions with heavy right-side tails (Figure 4I), the log-normal and gamma distributions were the best-fit models, whereas the modified ERF was less consistent, because the probabilities away from the mode followed a linear rather than a parabolic function on the semi-log plot. In such cases, the difference in the ΔAICc between the log-normal and gamma distributions was very close to zero, indicating equally plausible models (Table S2). The log-normal distribution was selected three times more often, however, than the gamma distribution as the best model. Hence, the log-normal distribution was generally a better model than the gamma distribution for the floral organ numbers. Our results suggest that the modeling of ovule development in *S*. *canadensis* will generate log-normal-like variation.

The organ-specific model selection can even occur independently of floral development. The number distribution of the seeds, which develop from ovules only after pollination, differed from that of the ovules: in *Hibiscus syricacus* (Malvaceae; data from Harris, 1911), the ovules tended to fit the modified ERF distribution, whereas the modified beta distribution was the best fit for the seeds (Figure 4J; Table 2). The transition between the different types of distributions from the ovules to the seeds might be caused during pollination. Ecological factors that cause variation in the distribution patterns of organ numbers would be an interesting avenue for further research.

#### 3.3.5. Ray florets

Although the ray floret is not a floral organ but itself a flower, it has similarity with a perianth organ in developmental and morphological aspects: they develop from the meristem and surround the compact inner organs. Historically, the Asteraceae ray florets were a main target of research on organ-number variation along with the Ranunculaceae floral organs (de Vries, 1894). Hence, it is worth examining the statistical model selection for the ray floret-number variation. The ERF was the dominant model for the ray florets (best fit for 29/43 data sets). The second dominant model was the modified beta distribution (5/43; e.g., *Leucanthemum vulgare*; Figure 4K), because it was the only one of the six models to explain bimodal distributions. It did not, however, seem to fit those distributions very well. For example, the organ number of the right peak is different between the measured data and the fitting function in Figure 4K. In addition, because the domain of organ number *X* in the modified beta distribution is defined between the two modes, the model cannot account for the organ-number distribution outside of the domain (i.e., less than the left mode or more than the right mode), suggesting a future problem in finding another model for the bimodality.

## 4. Discussion

### 4.1. Developmental bases of the modified ERF distribution

The modified ERF distribution requires three properties during floral development: (1) The concentric expression of homeotic genes, (2) A Gaussian distribution of the gene expression boundary, and (3) A semi-whorled arrangement. Here we discuss the biological bases of these three assumptions.

#### 4.1.1. The concentric gene expression during flower and inflorescence development

The identity of floral organs in *Arabidopsis thaliana* and *Antirrhinum majus* is determined by MADS-box genes expressed in concentric manner, which is known as the ABC model (Figure 3A). For example, we assumed that the number of sepals is determined by the expression boundary of gene B, as observed in Ranunculaceae (Gonçalves et al., 2013; see also a review by Soltis et al., 2007). Likewise, such concentric expression appears even in the inflorescences of the Asteraceae. The *TCP* family *CYC/DICH* genes, which determine the fates of the floret primordia, are expressed concentrically to the inflorescence apex in the radiate heads of the Asteraceae. For example, in *Gerbera hybrida*, the expression of *GhCYC2* follows a gradient along the radial axis of the inflorescence (Broholm et al., 2008). Similarly in *Senecio vulgaris, RAY1* and *RAY2*, the homologs of *GhCYC2*, are expressed in the outer floret primordia (Kim et al., 2008). Thus, the concentric expression of organ-fate determinants is widespread among eudicots, not only in flowers but also in inflorescences.

#### 4.1.2. The Gaussian distribution of the gene-expression boundary

Little is known about the stochastic variation in MADS-box and *TCP* gene-expression boundaries [*P_gene_*(*r*) in Equation 12], in contrast to the morphogen gradients in *Drosophila* embryogenesis (Houchmandzadeh et al., 2002; Bergmann et al., 2007). Although we assumed a Gaussian distribution for the variation, when the expression boundary follows other types of probability distributions (e.g., the log-normal or gamma distributions), the functional form of Equation 13 should be improved by integrating the probability (see Equation A1 in Supplementary Material). In addition to the noisy spatial patterns, the noisy temporal sequences of the fate determination gene expression (Alvarez-Buylla et al., 2010) could be another future problem for the floral organ number variation.

#### 4.1.3. The semi-whorled arrangement of the floral organs

Many Ranunculaceae species such as *Ranunculus* exhibit semi-whorled arrangements (Figure 3C; sometimes referred as false-whorls or pseudo-whorls) in their flowers (Schöffel, 1932). Even in some whorled flowers, the primordia initiation is sequential; in other words, their positional arrangement is transiently semi-whorled [e.g., Caryophyllaceae (Lyndon, 1978) and Oleaceae (Dadpour et al., 2011)]. In the Caryophyllaceae species *Silene coeli-rosa*, the positions of the primordia were measured quantitatively in the early stage of floral development, showing that the sepals took on a semi-whorled arrangement (Lyndon, 1978). If the ABC genes determine the organ fate at that stage, the modified ERF distribution is valid even for the whorled flowers.

### 4.2. Counter examples of the modified ERF distribution

**Figure 5:**
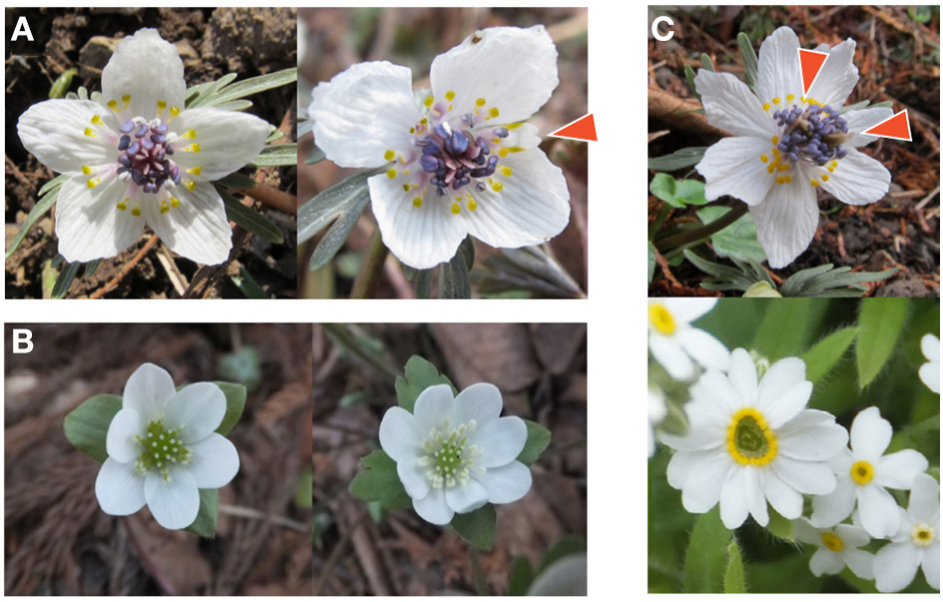
**Examples of flowers showing abnormal organ numbers**. **(A)** Homeotic transformation in *Eranthis pinnatifida* (Ranunculaceae). A typical flower with five tepals (left) and an abnormal flower with four tepals (right). The arrowhead indicates an organ with intermediate morphology between tepal and nectary. **(B)** Merosity variation in *Hepatica nobilis* (Ranunculaceae). The flower with three bracts has six tepals consisting of two trimerous (three-leaved) whorls (left), whereas the flower with four bracts has eight tepals consisting of two tetramerous (four-leaved) whorls (right). **(C)** Flower with large number of organs. An abnormal *E*. *pinnatifida* flower (top) has two clusters of pistils indicated by arrowheads, implying the fusion of two flowers. The abnormal flower of *Myosotis* sp. (Boraginaceae) with 13 petals has a skewed center, in contrast to the circular center of the normal flower with five petals (bottom).

We suggested a development-based model for the modified ERF distribution. The easiest way to confirm such a model based on homeotic stochasticity (Figure 5A) is to find a negative correlation between the organ numbers of successive whorls. Although the modified ERF distribution fit much of the observed floral organ variation well, there are some counter examples that do not exhibit such strong negative correlations. For example, there is no correlation between the numbers of tepals and nectaries in *Eranthis pinnatifida*. The absence of a negative correlation is partially explained by the MADS-box genes affecting not only the organ-identity determination but also the organ numbers, which the ABC model does not take into account: mutations in the ABC genes result in the partial loss of some organs or whorls [e.g., the partial or complete loss of the organs of the second and third whorls caused by mutations in the A gene, *APETALA2*, in *Arabidopsis thaliana* (Bowman et al., 1989; Kunst et al., 1989)]. In addition, merosity variation, where the numbers of sepals, petals, and stamens are strongly and positively correlated, was found in Ranunculaceae flowers (e.g., *Hepatica nobilis*; Figure 5B) and is common among Primulaceae flowers (e.g., *Trientalis europaea*; Tikhodeev and Tikhodeeva, 2001), indicating that homeotic variation is not the sole source of floral organ-number variation. Therefore, the variation should be represented by the sum of two or more distributions. Because the modified ERF distribution and the standard Gaussian distribution were equally selected as the best model for Primulaceae flowers (*Primula*×*julianna* and *T*. *europaea*; Table S3), we cannot distinguish between merosity and homeotic variation using the present framework. Further work should explore a model for merosity variation.

### 4.3. Future problems

#### 4.3.1. Improvement of the models

In some of the data sets for which the modified ERF distribution produced the lowest ΔAICc value, the right tail was rather closer to the log-normal distribution (Figure 4C), indicating that the best way to improve the overall model is to use the log-normal distribution for the expression boundary variation (Equation 12; Figure 3B). There is another possibility for the summation of two or more distributions caused by different developmental sources of stochasticity. One simple idea to confirm this possibility is to fit a limited range of the distribution, such as the right-tail and the left-tail separated by the mode, which would clarify whether the same or different laws govern decreases and increases, respectively, in the organ number relative to the mode.

We hardly discussed multimodal distributions, which have attracted some researchers who suggest that there is a relation between peak organ numbers and the Fibonacci series, especially among Asteraceae heads (Ludwig, 1895). There seem to be at least two different sources of multimodal distributions. One is stochastic changes in the number of whorls that differentiate into identical organs. Suppose that, for example, there are two whorls comprising eight and five organ primordia, respectively. If only the whorl with eight primordia differentiates into ray florets, there will be eight ray florets; whereas if both whorls become ray florets, there will be 13 ray florets. By assuming such multiple semi-whorls, the present modified ERF model could be improved for the multimodal distributions. The other source of multimodal distributions may be the fusion of the flowers, which is not accounted for by any of the present models. For example in *Myosotis* sp. (Boraginaceae), the mode of the petal number is five, but the distribution has a second peak at 10 (Table S1). The flowers with 10 petals seem to be generated by the fusion of two flowers (Figure 5C), which may be under hormonal control by cytokinins (Srinivasan and Mullins, 1979), suggesting that the examination of the fused flowers in the laboratory will give clues to how to construct a model for the multimodal distribution.

#### 4.3.2. Technical issues

The parameters after the convergence of the non-linear least-square method generally depend on the initial set of fitting parameters. Although we set the initial conditions intuitively to avoid divergent parameter fitting, there might be other initial conditions resulting better parameter sets (i.e., lower AICc). The fitting parameters were occasionally sensitive to initial conditions for the log-normal, the gamma, and the beta distributions when *p*-value is high (e.g., few asterisks in Table 1). As a result of the trials of several other initial conditions, we found that the best model changed in four out of 99 data sets of Ranunculaceae perianth organs (the best model for three data sets of *Ranunculus arvensis* petals changed to beta from the ERF, to standard Gaussian from beta, and to beta from the standard Gaussian, respectively; one data set of *R*. *ficaria* sepals changed to the ERF from beta) due to the change of fitting parameters for the beta distribution. We did not find any case that the best model changes depending on the initial conditions of the log-normal or the gamma distribution. A wide exploration of initial conditions could improve the selection of statistical models of organ-number variation.

### 4.4. Conclusion

The variation in the floral organ numbers of various eudicot species was fit to six statistical models. Statistical model selection revealed that the selection of the best model was reproducible for each species and organ. The modified ERF model, which we first proposed by assuming a semi-concentric arrangement of organ primordia following helical initiation and stochasticity in the concentric determination of organ fate during floral development, was widely selected for the perianth organs of eudicots, and even for the stamens and ray florets of some core eudicots. The standard Gaussian and log-normal distributions were selected, respectively, for the Oleaceae petals, which show the simultaneous initiation of the primordia, and the Papaveraceae ovules, which have a totally different developmental process compared with the perianth organs. We showed that the different distributions of morphological traits reflect different developmental processes. The modeling of developmental process of these organs and the statistical analyses of other species and organs will shed more light on the developmental and evolutionary sources of morphological variation.

## Author contributions

Conceived and designed the experiments: Miho S. Kitazawa, Koichi Fujimoto. Performed the data collection and the statistical analysis: Miho S. Kitazawa, Koichi Fujimoto. Analyzed the data: Miho S. Kitazawa, Koichi Fujimoto. Contributed materials/analysis tools: Miho S. Kitazawa, Koichi Fujimoto. Wrote the paper: Miho S. Kitazawa, Koichi Fujimoto.

### Conflict of interest statement

The authors declare that the research was conducted in the absence of any commercial or financial relationships that could be construed as a potential conflict of interest.
