# Supplementary material for: A developmental basis for stochasticity in floral organ numbers
Source: Front Plant Sci. 2014 Nov 3;5:545. doi: 10.3389/fpls.2014.00545 (PMC4217355; doi:10.3389/fpls.2014.00545)
Supplement: Supplementary file 2 [file Presentation1.PDF]

A DEVELOPMENTAL BASIS FOR STOCHASTICITY IN FLORAL ORGAN NUMBERS

Miho S. Kitazawa and Koichi Fujimoto

http://www.frontiersin.org/journal/10.3389/fpls.2014.00545/abstract

SUPPLEMENTARY MATERIAL

DERIVATION OF ERROR FUNCTION

When the expression boundary of a homeotic gene is located between the X-th and $X+1$-th floral organ primordia, the number of the organs that differentiate into the outer whorl identity (e.g., sepal in Figure 3) becomes X. The probability of the organ number being X is calculated by integrating the probability of the boundary position given by Eq. 12 for this region

$$P_{er}(X) = \int_{r_{X+1}}^{r_X} P_{gene}(r)\, dr. \tag{A1}$$

where r_{X+1} and r_X are the radial positions of the $X+1$-th and X-th primordia, respectively (Figure 3C). The positions are rewritten as follows by the four parameters of the floral whorl: μ_r (the average boundary position), ex (the radial distance between μ_r and the interior edge of the exterior whorl), in (the radial distance between μ_r and the exterior edge of the interior whorl), and d (the radial distance between two successive primordia within each whorl)

$$r_X = \begin{cases} \mu_r + ex + d(Mo - X) & (X \leq Mo) \\ \mu_r - in + d(Mo - X + 1) & (X > Mo), \end{cases} \tag{A2}$$

where Mo is the mode defined by the most frequent number of floral organs (e.g., $Mo = 5$ for sepals in Figure 3B). By substituting Eq. A2 into Eq. A1, the probability of sepal number X becomes

$$P_{er}(X) = \begin{cases} \int_{\mu_r+ex+d(Mo-X-1)}^{\mu_r+ex+d(Mo-X)} P_{gene}(r)\, dr & (X < Mo) \\ \int_{\mu_r-in+d(Mo-X)}^{\mu_r-in+d(Mo-X+1)} P_{gene}(r)\, dr & (X > Mo) \\ \int_{\mu_r-in+d(Mo-X)}^{\mu_r+ex+d(Mo-X)} P_{gene}(r)\, dr & (X = Mo). \end{cases} \tag{A3}$$

Using Eq. 12 and the variable transformation $z = (r - \mu_r)/\sqrt{2}\sigma_r$, Eq. A3 is rewritten as

$$P_{er}(X) = \begin{cases} \frac{1}{\sqrt{\pi}} \int_{\frac{ex+d(Mo-X-1)}{\sqrt{2}\sigma_r}}^{\frac{ex+d(Mo-X)}{\sqrt{2}\sigma_r}} \exp\left(-z^2\right) dz & (X < Mo) \\ \frac{1}{\sqrt{\pi}} \int_{\frac{\mu_r-in+d(Mo-X)}{\sqrt{2}\sigma_r}}^{\frac{\mu_r-in+d(Mo-X+1)}{\sqrt{2}\sigma_r}} \exp\left(-z^2\right) dz & (X > Mo) \\ \frac{1}{\sqrt{\pi}} \int_{\frac{-in}{\sqrt{2}\sigma_r}}^{\frac{ex}{\sqrt{2}\sigma_r}} \exp\left(-z^2\right) dz & (X = Mo) \end{cases} \tag{A4}$$

The Gaussian integral with finite range is rigorously represented by the error function (ERF) given by Eq. 14. By substituting Eq. 14 into Eq. A4 and normalizing parameters as

$$ex_d = \frac{ex}{d}, in_d = \frac{in}{d}, \sigma_d = \frac{\sigma}{d}, \tag{A5}$$

we obtain Eq. 13.

TABLE S1.

The Excel datasheet including all data of floral organ numbers we used for the present analysis. The sheet "Published data" contains the data from authors listed in the file, whereas "Original data" contains the data collected by ourselves.

TABLE S2.

The ΔAICc values of the fittings for floral organ numbers of *Sanguinaria canadensis*. Data from **Spencer** (1944)(petals) and **Harris** (1910) (ovules and seeds). The p-values of the parameters are: *** < 0.01, ** < 0.05, * < 0.1.

Organ	n	Gauss	LogNormal	Gamma	Beta	Poisson	ERF
petals	964	*** 33.59	** 18.06	19.25	89.87	* 14.93	*** 0.00
petals	1994	*** 21.59	** 18.37	*** 18.73	22.65	** 18.40	*** 0.00
petals	2470	*** 38.50	** 39.14	*** 39.59	30.38	*** 31.94	*** 0.00
petals	5002	*** 34.74	29.39	* 38.52	9.52	*** 28.64	*** 0.00
petals	2371	*** 23.80	* 21.99	*** 23.44	32.66	*** 13.04	*** 0.00
petals	5000	*** 48.72	44.78	*** 41.99	*** 0.00	*** 30.14	*** 4.97
petals	5332	*** 75.37	** 74.01	*** 75.11	*** 45.20	*** 58.17	*** 0.00
petals	5160	*** 33.88	*** 23.38	17.72	22.78	*** 18.40	*** 0.00
petals	2916	*** 29.20	26.06	*** 31.61	78.98	*** 15.26	*** 0.00
ovules	1000	*** 22.85	*** 0.16	*** 0.00	2.96	*** 0.40	25.44
ovules	1000	*** 34.80	*** 0.00	*** 1.10	4.01	*** 3.16	37.08
ovules	1000	*** 50.00	*** 0.00	*** 5.75	117.43	*** 33.08	*** 39.70
ovules	1000	*** 53.74	*** 0.00	*** 1.33	4.01	*** 38.64	*** 49.70
ovules	1000	*** 43.26	*** 0.00	*** 2.56	4.96	*** 22.80	*** 44.63
ovules	1000	*** 79.93	*** 0.00	*** 9.95	22.64	*** 68.48	*** 71.38
ovules	400	*** 35.27	*** 7.49	0.00	0.86	*** 20.32	*** 16.01
ovules	400	*** 25.69	*** 0.00	5.23	8.86	*** 11.36	* 26.12
ovules	400	*** 23.55	*** 0.00	*** 3.95	6.86	*** 9.84	20.81
ovules	400	*** 14.97	0.86	** 3.11	5.96	*** 2.42	0.00
ovules	400	*** 40.42	*** 0.00	*** 12.00	14.65	*** 27.97	*** 31.50
ovules	400	*** 20.07	9.61	** 11.01	13.39	*** 14.48	0.00
seeds	1000	*** 26.10	0.00	4.64	7.38	*** 4.57	23.73
seeds	1000	*** 18.22	0.00	1.98	4.72	2.61	*** 4.20
seeds	1000	*** 30.00	0.00	*** 3.65	5.96	*** 16.59	28.88
seeds	400	*** 1.59	3.29	3.41	6.35	*** 2.62	0.00
seeds	400	*** 3.29	4.86	4.92	7.77	*** 5.15	*** 0.00
seeds	400	*** 19.85	20.99	21.12	23.55	*** 18.88	*** 0.00

TABLE S3.

The ΔAICc values of the fittings for floral organ numbers of Primulaceae flowers. Pr.ju *Primula×julianna*, Tr.eu *Trientalis europaea* (**Charlier**, 1913; **Matthews and Roger**, 1941; **Tikhodeev and Tikhodeeva**, 2001). The p-values of the parameters are: *** < 0.01, ** < 0.05, * < 0.1.

Species	Organ	n	Gauss	LogNormal	Gamma	Beta	Poisson	ERF
Pr.ju	stamens	603	*** 0.00	7.66	** 7.85	13.95	*** 58.18	*** 5.44
Pr.ju	petals	603	*** 0.00	10.74	11.57	18.80	** 73.03	*** 6.05
Pr.ju	petals	475	*** 7.99	4.25	4.98	NA	* 7.04	** 0.00
Pr.ju	sepals	603	*** 0.00	9.45	12.44	94.95	** 74.52	*** 7.65
Tr.eu	stamens	2370	*** 0.00	19.47	29.16	NA	31.43	*** 5.32
Tr.eu	stamens	2618	*** 13.89	35.78	36.42	** NA	46.76	*** 0.00
Tr.eu	stamens	173	*** 0.00	28.55	25.90	NA	30.70	15.29
Tr.eu	petals	2370	*** 0.00	19.47	29.16	NA	31.43	*** 5.32
Tr.eu	petals	2618	*** 0.38	35.51	35.27	NA	46.95	*** 0.00
Tr.eu	sepals	2370	*** 0.00	19.47	29.16	NA	31.43	*** 5.32
Tr.eu	sepals	2618	*** 20.38	47.17	39.68	*** 28.47	64.38	*** 0.00

TABLE S4.

The ΔAICc values of the fittings represented in Figure 4. The first and second lowest values are shaded by dark and light grey, respectively. The p-values of the parameters are: *** < 0.01, ** < 0.05, * < 0.1.

Figure	n	Gauss	LogNormal	Gamma	Beta	Poisson	ERF
A	1033	*** 29.72	** 21.32	** 18.78	NA	*** 7.30	*** 0.00
B	252	*** 7.86	11.99	15.59	22.05	** 19.71	*** 0.00
C	3000	*** 31.75	** 12.09	*** 15.92	20.64	*** 26.05	*** 0.00
D	222	*** 27.84	13.99	*** 10.00	37.61	*** 0.00	** 12.04
E	5332	*** 75.37	** 74.01	*** 75.11	*** 45.20	*** 58.17	*** 0.00
F	NA	*** 17.12	* 21.59	** 18.97	NA	*** 0.00	21.29
G	632	*** 26.35	34.14	34.14	56.22	50.01	*** 0.00
H	5162	*** 0.00	10.11	* 11.20	NA	24.10	*** 11.36
I	1000	*** 79.93	*** 0.00	*** 9.95	22.64	*** 68.48	*** 71.38
J	1594	*** 44.31	47.54	47.83	0.00	*** 51.76	*** 30.39
K	700	*** 13.06	*** 5.59	0.84	*** 0.00	12.48	*** 1.04
